# Macrophage migration inhibitory factor has a permissive role in concanavalin A-induced cell death of human hepatoma cells through autophagy

**DOI:** 10.1038/cddis.2015.349

**Published:** 2015-12-03

**Authors:** Y-C Lai, Y-C Chuang, C-P Chang, T-M Yeh

**Affiliations:** 1 The Institute of Basic Medical Sciences, College of Medicine, National Cheng Kung University, Tainan, Taiwan; 2 Department of Medical Laboratory Science and Biotechnology, College of Medicine, National Cheng Kung University, Tainan, Taiwan; 3 Department of Microbiology and Immunology, College of Medicine, National Cheng Kung University, Tainan, Taiwan

## Abstract

Concanavalin A (ConA) is a lectin and T-cell mitogen that can activate immune responses. In recent times, ConA-induced cell death of hepatoma cells through autophagy has been reported and its therapeutic effect was confirmed in a murine *in situ* hepatoma model. However, the molecular mechanism of ConA-induced autophagy is still unclear. As macrophage migration inhibitory factor (MIF), which is a proinflammatory cytokine, can trigger autophagy in human hepatoma cells, the possible involvement of MIF in ConA-induced autophagy was investigated in this study. We demonstrated that cell death is followed by an increment in MIF expression and secretion in the ConA-stimulated human hepatoma cell lines, HuH-7 and Hep G2. In addition, ConA-induced autophagy and cell death of hepatoma cells were blocked in the presence of an MIF inhibitor. Knockdown of endogenous MIF by small hairpin RNA confirmed that MIF is required for both ConA-induced autophagy and death of hepatoma cells. Furthermore, signal pathway studies demonstrated that ConA induces signal transducer and activator of transcription 3 (STAT3) phosphorylation to trigger MIF upregulation, which in turn promotes Bcl-2/adenovirus E1B 19 kDa-interacting protein 3 (BNIP3)-dependent autophagy. By using a murine *in situ* hepatoma model, we further demonstrated that MIF contributes to anti-hepatoma activity of ConA by regulating STAT3–MIF–BNIP3-dependent autophagy. In summary, our findings uncover a novel role of MIF in lectin-mediated anti-hepatoma activities by regulating autophagy.

Autophagy is a ‘self-digestion' sequential process that regulates the turnover of intracellular organelles and macromolecules. This process begins with lipidation of cytosolic microtubule-associated protein light chain 3 (LC3-I) with phosphatidylethanolamine to form conjugated LC3-II, which is involved in the formation of double-membrane-bound autophagosome. At the late stage, the double-membrane autophagosomes fuse with lysosomes to form autophagolysosomes, which mediate the degradation of their contents. Autophagy has important roles in the homeostatic mechanisms that balance energy resources and degrade dysfunctional proteins, damaged organelles and intracellular pathogens, which enables the cell to survive under stress.^1^ Autophagy is also involved in the pathogenesis of many diseases, including cancer.^2, 3, 4^ However, the role of autophagy in cancer is similar to a double-edged sword that depends on tumor types and stages.^5, 6, 7^ Autophagy might be essential for maintaining cell survival that promotes the growth of tumors. In contrast, many studies have shown that there is excessive or abnormal autophagy activity in some tumor cells, such as hepatoma and breast cancer cells,^8, 9, 10^ which may contribute to autophagic cell death and thus limit tumor burden.^11^ Because of the contrasting properties of autophagy concerning its effects on tumor progression and suppression, the roles of autophagy in tumorigenesis and cancer progress remain controversial.

Concanavalin A (ConA) is a plant lectin that was originally extracted from jack bean, *Canavalia ensiformis.* ConA can specifically bind to certain terminal sugars such as α-D-mannoside or methyl α-D-mannopyranoside in blood cells, and it can bind to some immunoglobulins and lipoproteins.^12^ The ability of lectins such as ConA to bind to cell surfaces depends on the degree and forms of glycosylation for different cells.^13, 14^ In particular, in tumor cells, different levels of glycosylation are found in different cells, which make the tumor cells preferentially sensitized to bind certain lectins.^15^ On the other hand, ConA is also a T-cell mitogen that can activate the immune system, recruit lymphocytes and elicit cytokine production.^16, 17, 18^ Hepatoma or hepatocellular carcinoma (HCC) is the sixth most common solid tumor and the third leading cause of cancer-related death, but appropriate treatment is still lacking.^19^ Therefore, new therapeutic strategies for advanced stage HCC are necessary to provide better outcome prediction. As ConA possesses both immunomodulatory and cytotoxic activities against hepatoma cells, it has the potential to be a new anti-hepatoma therapeutic agent.^20, 21^ In our previous study, we found that ConA has a therapeutic effect in a murine *in situ* hepatoma model by arousing a strong immune response against tumor growth and inducing hepatoma cell death through autophagy *in vitro*.^22, 23, 24^ However, the detailed mechanism of ConA-induced autophagic cell death of hepatoma cells remains unclear.

Macrophage migration inhibitory factor (MIF) was first identified as a cytokine that is released from T cells and can inhibit the random migration of macrophages.^25, 26^ MIF is an evolutionarily highly conserved molecule that contains 114 amino acids to form a 12.5-kDa protein.^27^ MIF exists as a trimer and consists of three identical subunits that possess unique catalytic functions as a tautomerase.^28^ In addition to T cells, MIF is widely distributed in different cells, such as epithelial cells, endothelial cells, hepatocytes and even cancer cells.^29, 30^ Despite its wide distribution, MIF secretion is tightly regulated under stress conditions. After binding to its receptor CD74, MIF activates downstream pathways in autocrine or paracrine manners.^31, 32^ Secreted MIF is capable of modulating both innate and adaptive immune responses.^30, 33^ In addition, MIF has important roles in tumor growth, metastasis and tumor-associated angiogenesis.^34, 35^

Numerous studies have revealed that ConA can elicit the secretion of pro-inflammatory cytokines, including MIF.^36, 37, 38, 39^ Studies have also reported that ConA can induce hepatitis in mice, and that the knockdown of MIF can protect mice against ConA-induced liver injury.^40^ However, the involvement of MIF in ConA-induced autophagic cell death of hepatoma cells is unclear. In recent times, we found that MIF can induce autophagy through reactive oxygen species formation in hepatoma cells.^41^ Therefore, in this study, we proposed that MIF is involved in the ConA-induced autophagy and cell death of human hepatoma cells both *in vitro* and *in vivo*. Our results indicate that MIF is required for ConA-induced autophagic cell death of hepatoma cells and anti-tumor activities in mice.

## Results

### ConA triggers autophagic flux in human hepatoma cells

**Figure 1:**
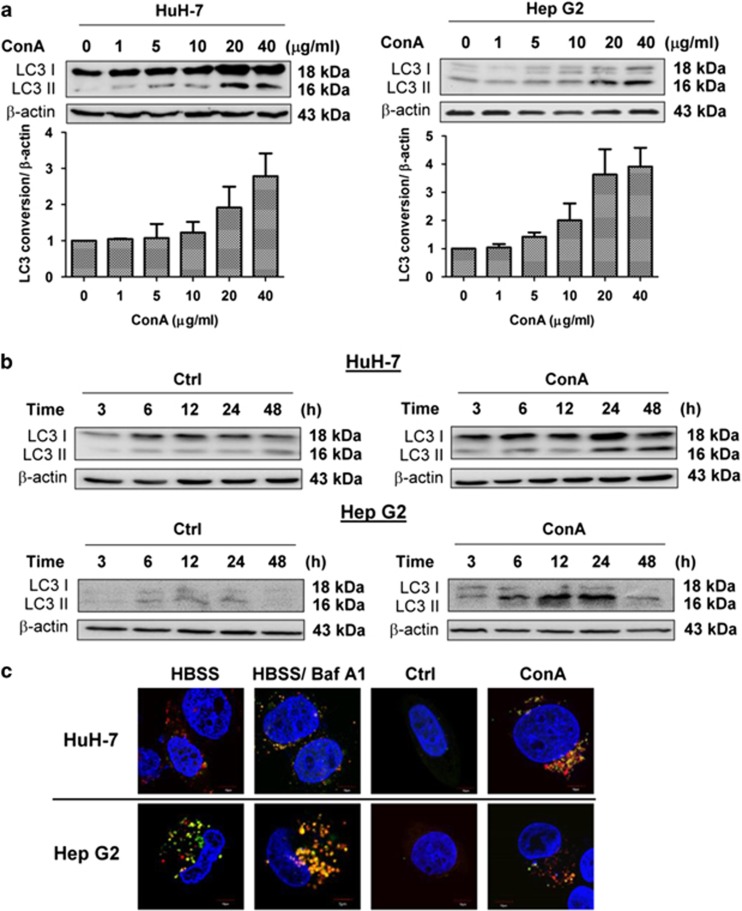
ConA induces autophagy in human hepatoma cells. (**a**) HuH-7 and Hep G2 cells were treated with the indicated doses of ConA for 24 h. Quantification of LC3 conversion is presented as the fold increase of LC3-II compared with the control cells after normalization to the corresponding *β*-actin levels (**b**) HuH-7 and Hep G2 cells were treated with or without ConA (20 *μ*g/ml) for the indicated time points. LC3 conversion was determined using western blotting. (**c**) HuH-7 and Hep G2 transfected with ptfLC3 plasmid were treated with or without ConA (20 *μ*g/ml) for 24 h. As serum-starvation control, HuH-7 and Hep G2 cells were treated with HBSS in the presence or absence of BafA1 (10 nM) for 1 h. The images were taken by an Olympus FV-1000 MPE confocal microscope. Scale bar represents 10 *μ*m. FV10-ASW 4.0 Viewer software was used to merge green (EGFP) and red (mRFP) images to detect synaptic LC3 puncta (yellow). The blue staining indicated the nucleus. All data are presented as the means±S.D. from at least triplicate independent experiments

Studies have shown that ConA can trigger autophagy and induce an autophagic-like cell death in ML-1, which is a murine hepatoma cell line.^24^ We first confirmed that ConA-induced autophagic flux in two types of human hepatoma cell lines: HuH-7 and Hep G2 (Figure 1). Both protein and western blot staining showed an increase in LC3-II in a dose- and time-dependent manner in these two human hepatoma cell lines after ConA stimulation (Figures 1a and b). Furthermore, HuH-7 and Hep G2 cells transfected with a tandem fluorescent-tagged LC3 (ptfLC3) plasmid were used to define the stage of the autophagosome. As EGFP is more sensitive to low pH levels than mRFP is, the EGFP signal decayed in the acidic environment of lysosomes, whereas the mRFP signal remained steady. As a result, the reduction of green puncta may indicate the conversion of an autophagosome to an autophagolysosome.^42^ As shown in Figure 1c, after 24 h of ConA treatment, LC3 puncta increased both in HuH-7 and Hep G2 cells, and some of these puncta started to turn red, which indicated the decomposition of EGFP. Therefore, active autophagic flux was triggered in ConA-treated HuH-7 cells.

### ConA triggers autophagic but not apoptotic cell death in human hepatoma cells

**Figure 2:**
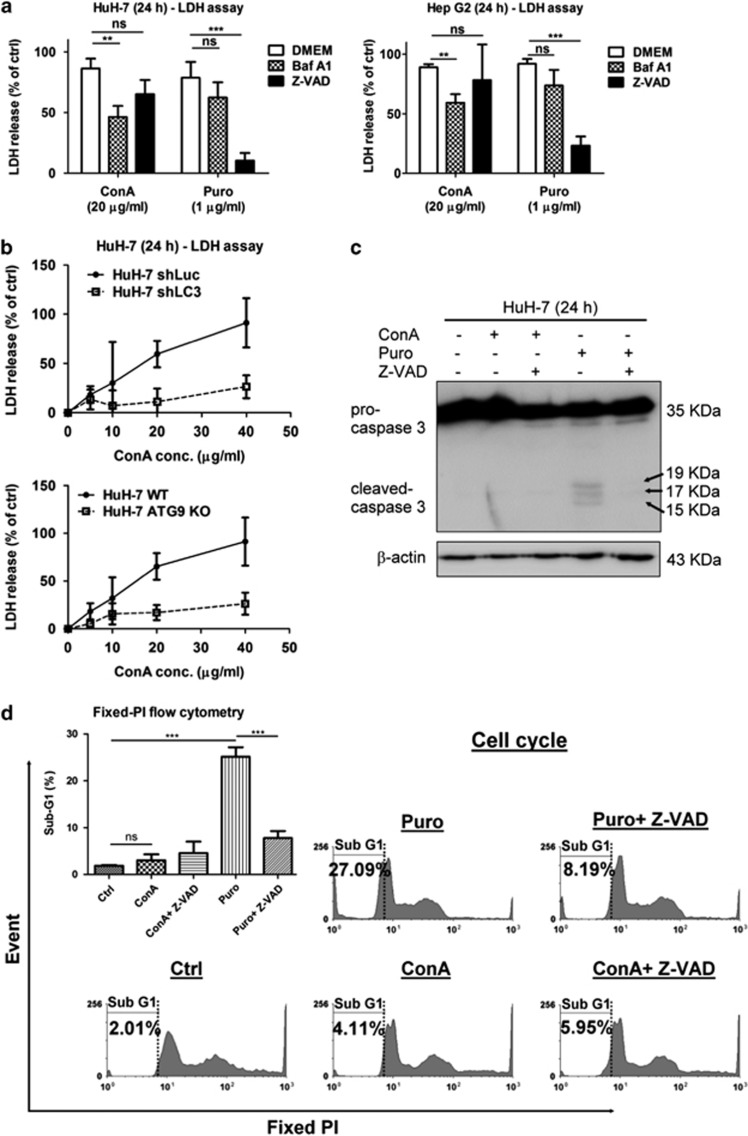
ConA triggers autophagic but not apoptotic cell death in human hepatoma cells. (**a**) HuH-7 and Hep G2 cells were treated with ConA (20 *μ*g/ml) or Puro (1 *μ*g/ml) in the presence or absence of either Baf A1 (1 nM) or Z-VAD (20 *μ*M) for 24 h. After treatment, cytotoxicity was analyzed by LDH release assay. (**b**) HuH-7-shLuc, HuH-7-shLC3, HuH-7 wild-type (WT) and HuH-7 ATG9 knockout (KO) cells were treated with different doses of ConA as indicated for 24 h. After treatment, cytotoxicity was analyzed using an LDH release assay (**c**) HuH-7 cells were treated with ConA (20 *μ*g/ml) or Puro (1 *μ*g/ml) in the presence or absence of Z-VAD (20 *μ*M) for 24 h. Cell lysates were subjected to detect pro-caspase 3 and cleaved-caspase 3. (**d**) HuH-7 cells were treated with ConA (20 *μ*g/ml) or Puro (1 *μ*g/ml) in the presence or absence of Z-VAD (20 *μ*M) for 24 h. Sub-G1 phase was performed by fixed PI staining and analyzed using flow cytometry. Quantification of sub-G1 phase was analyzed using the WinMDI 2.9 software (the upper left). All data are presented as the mean±S.D. from at least triplicate independent experiments; *P<0.05, **P<0.01, ***P<0.001, ns indicates no significance

To study the type of cell death induced by ConA, we compared the cytotoxicity induced by ConA or puromycin (Puro), an apoptotic inducer using LDH release assay in the presence of bafilomycin A1 (Baf A1), an autolysosomal fusion inhibitor that inhibits autophagy process or VAD-FMK (Z-VAD), a pan caspase inhibitor that inhibits classical apoptosis pathway (Figure 2). As high concentrations of Baf A1 (≥10 nM) decrease cell viability after 24 h of incubation, we chose to use Baf A1 at 1 nM for this experiment.^43^ As shown in Figure 2a, both ConA and Puro induced LDH release from hepatoma cells. However, in the presence of Baf A1 but not Z-VAD, the cytotoxicity of ConA to hepatoma cells was significantly inhibited. On the other hand, Z-VAD but not Baf A1 could significantly inhibit Puro-induced hepatoma cell death. LC3 and ATG9 deficiency in cells (Figure 2b and Supplementary Data) further confirms that the ConA-induced cytotoxicity of hepatoma cells is autophagy dependent. In addition, Puro but not ConA significantly induced the caspase 3 activation of hepatoma cells (Figure 2c). DNA fragment analysis by fixed PI staining using flow cytometry also indicated that the increase in sub-G1 phase observed in Puro- but not ConA-treated hepatoma cells (27.09 *versus* 4.11%, respectively; Figure 2d and Supplementary Data). Taken together, these results suggested that ConA induced autophagic cell death but not apoptosis in human hepatoma cells.

### ConA triggers MIF expression and secretion in human hepatoma cells

**Figure 3:**
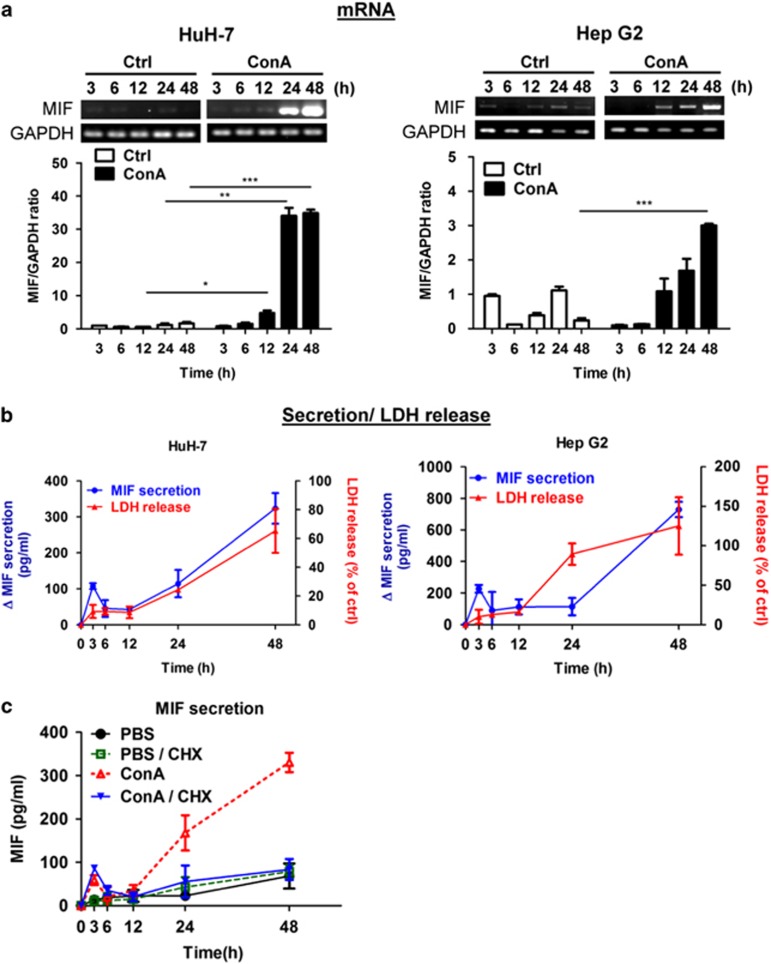
ConA triggers MIF expression and secretion in human hepatoma cells. HuH-7 and Hep G2 cells were treated with or without ConA (20 *μ*g/ml) for indicated time periods. (**a**) The MIF mRNA level was determined by RT-PCR. (**b**) The level of MIF secretion from culture supernatant was detected by MIF-ELISA (left *y* axis). △MIF represents MIF concentration in ConA-treated media compared with the control group. The cytotoxicity was analyzed by LDH release activity from culture supernatant (right *y* axis). LDH release (% of ctrl) represents the percentage of LDH activity in media compared with the control group. (**c**) HuH-7 cells were treated with or without ConA (20 *μ*g/ml) for indicated time points in the presence or absence of cycloheximide (CHX) (10 *μ*M) pre-treatment for 30 min. The level of MIF from culture supernatant was detected by MIF-ELISA. All data are presented as the mean±S.D. from at least triplicate independent experiments; *P<0.05, **P<0.01, ***P<0.001, ns indicates no significance

As MIF can trigger autophagy in human hepatoma cells, we tested whether MIF is involved in ConA-induced autophagic cell death. Following ConA stimulation, MIF mRNA expression was increased in time- and dose-dependent manners in both HuH-7 and Hep G2 cell lines, as determined by reverse transcription PCR (RT-PCR) (Figure 3a and Supplementary Data). MIF protein secretion and cytotoxicity were also increased after ConA treatment in dose- and time-dependent manners in HuH-7 and Hep G2 cells (Figure 3b and Supplementary Data). Moreover, we found that MIF secretion first peaked at 3 h after ConA treatment. As shown in Figure 3c, this peak of MIF secretion was not inhibited in the presence of cycloheximide at 3 h (a protein synthesis inhibitor) and may represent the release of pre-formed MIF from the cytosol. Thus, ConA stimulation could trigger the secretion of pre-formed cytosolic MIF at 3 h and the subsequent *de novo* synthesis of MIF.

### MIF has a permissive role in ConA-induced cell death in human hepatoma cells

**Figure 4:**
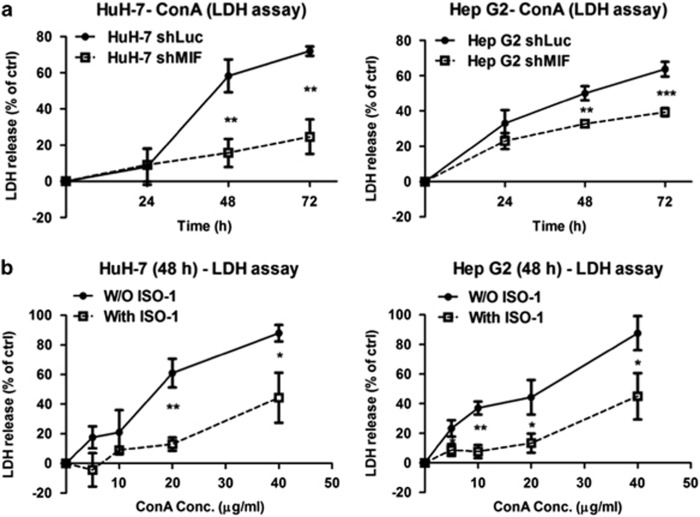
MIF participates in ConA-induced cell death in human hepatoma cells. (**a**) HuH-7-shLuc/shMIF and Hep G2-shLuc/shMIF cells were treated with or without ConA (20 *μ*g/ml) for the indicated time periods. (**b**) HuH-7 and Hep G2 cells were treated with different doses of ConA as indicated in the presence or in the absence of ISO-1 (50 *μ*M) for 48 h. The cytotoxicity was analyzed by LDH release activity from culture supernatant. LDH release (% of ctrl) represents the percentage of LDH activity in media compared with the control group

To further confirm the requirement of MIF in ConA-induced cell death of hepatoma cells, we generated MIF knockdown cell lines of HuH-7 and Hep G2 using short hairpin RNA (shRNA; Supplementary Data). As shown in Figure 4a, ConA-induced cell death was abrogated in the cells that lacked endogenous MIF compared with the control cells (knockdown of Luciferase). In addition, the inhibition of MIF activity by a MIF inhibitor, (S,R)-3-(4-hydroxyphenyl)-4,5-dihydro-5-isoxazole acetic acid (ISO-1), attenuated ConA-induced cell death in both HuH-7 and Hep G2 cells (Figure 4b). These data indicated that MIF is required for ConA-triggered cytotoxicity in human hepatoma cells.

### MIF participates in ConA-induced autophagy of human hepatoma cells

**Figure 5:**
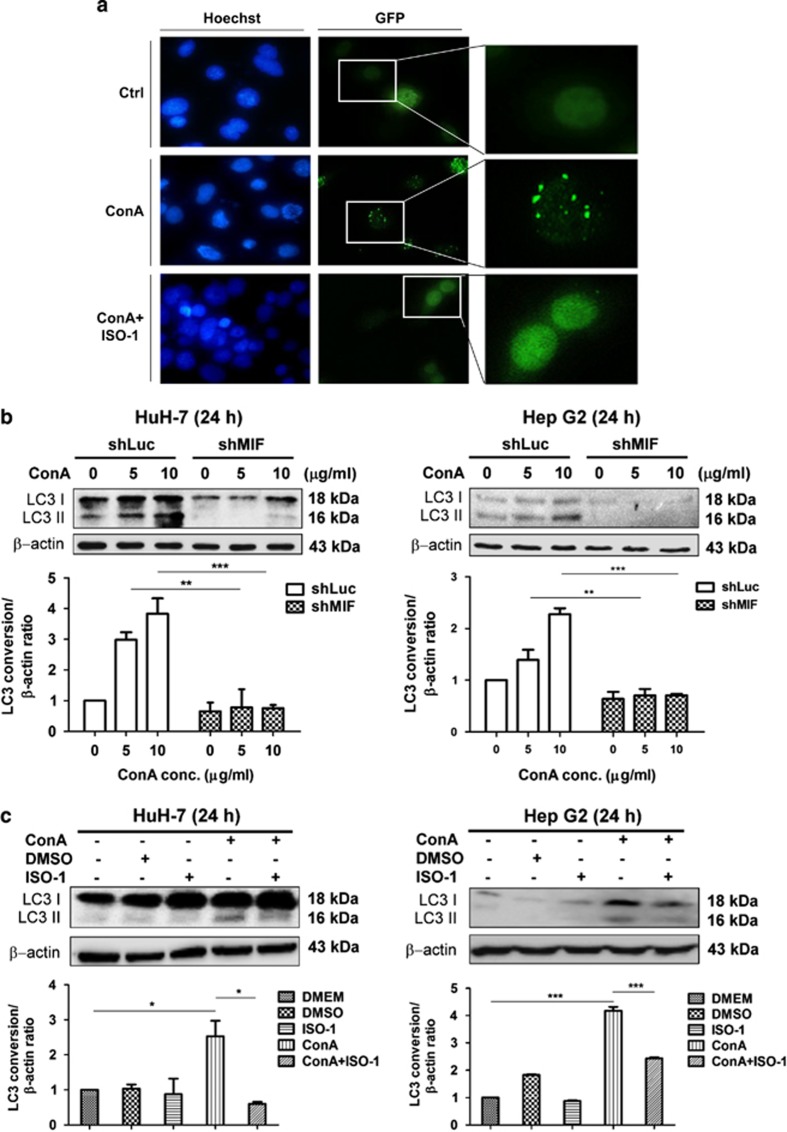
MIF participates in ConA-induced autophagy of human hepatoma cells. (**a**) HuH-7 cells transfected with pEGF-LC3 plasmid were treated with ConA (20 *μ*g/ml) in the presence or in the absence of ISO-1 (50 *μ*M) for 24 h. The images were taken by fluorescence microscopy at a magnification of × 400 and × 600. Hoechst and GFP represents the nucleus and LC3 punctae, respectively. (**b**) HuH-7-shLuc/shMIF and Hep G2-shLuc/shMIF cells were treated with ConA at different doses as indicated for 24 h. Cell lysates were subjected to detect LC3 conversion by western blotting. (**c**) HuH-7 and Hep G2 cells were treated with ConA (20 *μ*g/ml) in the presence or in the absence of ISO-1 (50 *μ*M) for 24 h. Cell lysates were subjected to detect LC3 conversion by western blotting. Quantification of LC3 conversion is presented as the fold increase of LC3-II compared with the control cells after normalization to the corresponding *β*-actin levels. All data are presented as the mean±S.D. from at least triplicate independent experiments; *P<0.05, **P<0.01, ***P<0.001, ns indicates no significance

As ConA induced hepatoma cell death through autophagy, we next examined whether MIF regulates ConA-induced cell death via autophagy. As expected, by analyzing LC3 punctae in fluorescence microscope, we found that ISO-1 attenuated ConA-triggered autophagy formation (Figure 5a). In addition, silencing of the MIF gene or inhibition of MIF activity by ISO-1 led to an attenuation of LC3-II conversion, as measured by western blotting (Figures 5b and c). These data suggested that MIF indeed has important roles in ConA-induced autophagy formation in human hepatoma cells.

### ConA internalization is required to induce STAT3–MIF–BNIP3-mediated autophagy in hepatoma cells

**Figure 6:**
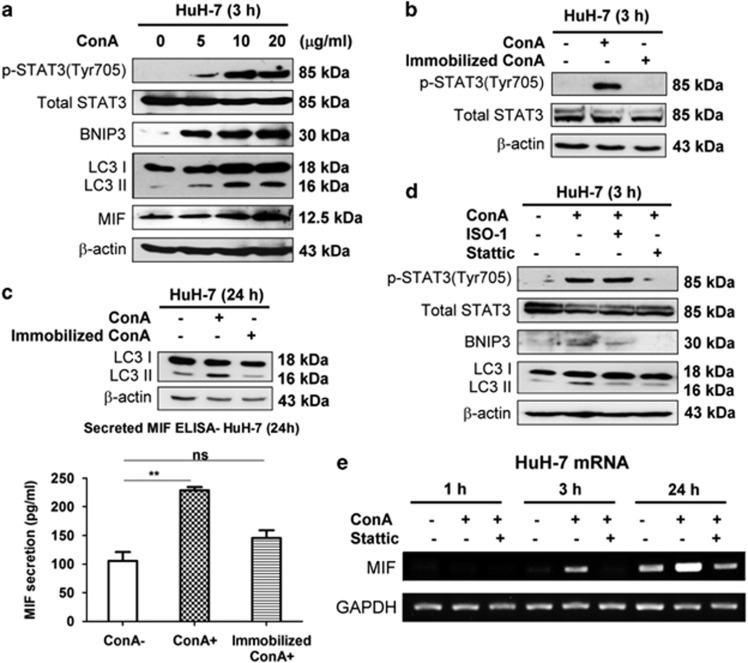
ConA is internalized to induce STAT3–MIF–BNIP3-mediated autophagy in hepatoma cells. (**a**) HuH-7 cells were treated with ConA at different dose as indicated at 3 h. The lysates were subjected to detect MIF expression, phosphorylation of STAT3 (Tyr705), total STAT3, BNIP3 induction and LC3 conversion by western blotting. (**b**) HuH-7 cells were treated with soluble ConA (20 *μ*g/ml) or beaded-agarose ConA (20 *μ*g/ml) for 3 h. Cell lysates were subjected to detect phosphorylation of STAT3 (Tyr705) and total STAT3 by western blotting. (**c**) HuH-7 cells were treated with soluble ConA (20 *μ*g/ml) or beaded-agarose ConA (20 *μ*g/ml) for 24 h. Cell lysates were subjected to detect LC3 conversion by western blotting (up panel). The amount of MIF secretion in supernatant was determined by MIF-ELISA (bottom panel). (**d**) HuH-7 cells were treated with ConA (20 *μ*g/ml) in the presence of either Stattic (10 *μ*g/ml) or ISO-1 (50 *μ*M) for 3 h. Cell lysates were subjected to detect phosphorylation of STAT3 (Tyr705), total STAT3, BNIP3 induction and LC3 conversion by western blotting. (**e**) HuH-7 cells were treated with ConA (20 *μ*g/ml) in the presence or absent of Stattic (10 *μ*g/ml) during different time periods as indicated. The MIF mRNA level was determined by RT-PCR

Bcl-2/adenovirus E1B 19 kDa-interacting protein 3 (BNIP3), a mitochondria-related protein, has been suggested to be involved in the ConA-induced autophagic cell death of hepatoma cells.^24^ Furthermore, a recent study showed that ConA triggers BNIP3-mediated autophagy via the Janus kinase (JAK)/signal transducer and activator of transcription 3 (STAT3) signaling pathway in mesenchymal stromal cells and glioblastoma cells.^44, 45^ In addition, STAT3 is activated through the phosphorylation of tyrosine 705 in response to several cytokines. Therefore, we examined whether STAT3 and BNIP3 are involved in the ConA-induced autophagy of hepatoma cells. As shown in Figure 6a, not only MIF expression and LC3-II formation but also STAT3 phosphorylation and BNIP3 induction were increased in a dose-dependent manner after ConA treatment. As ConA internalization into cells is required to trigger autophagy in hepatoma cells,^24^ we next tested whether ConA internalization was also required to induce MIF secretion and thereby facilitate autophagy formation. We found that only soluble ConA, but not immobilized ConA, triggered STAT3 phosphorylation and LC3-II formation (Figures 6b and c). To further understand the sequential events induced by ConA-stimulated hepatoma cells, we used Stattic (STAT3 activation inhibitor) and found that it suppressed STAT3 phosphorylation, BNIP3 induction and LC3-II conversion (Figure 6d). However, the inhibition of MIF by its inhibitor (ISO-1) only abolished ConA-triggered BNIP3 expression and autophagy but not phosphorylation of STAT3 (Figure 6d). These results suggested that MIF may be upstream of BNIP3-mediated autophagy and downstream of STAT3. Indeed, treating hepatoma cells with recombinant human MIF (rMIF) directly induced BNIP3 expression but not STAT3 phosphorylation, which were activated by ConA (Supplementary Data). The requirement of STAT3 activation for ConA-induced MIF expression was further confirmed by the suppression of STAT3 phosphorylation using Stattic. As shown in Figure 6e, in the presence of Stattic, ConA-induced MIF mRNA expression was significantly blocked. Taken together, our results suggest that ConA internalization induces STAT3 phosphorylation, which leads to MIF secretion and BNIP3-dependent autophagy.

### Suppression of MIF abrogates the therapeutic effect of ConA on hepatoma *in vivo*

**Figure 7:**
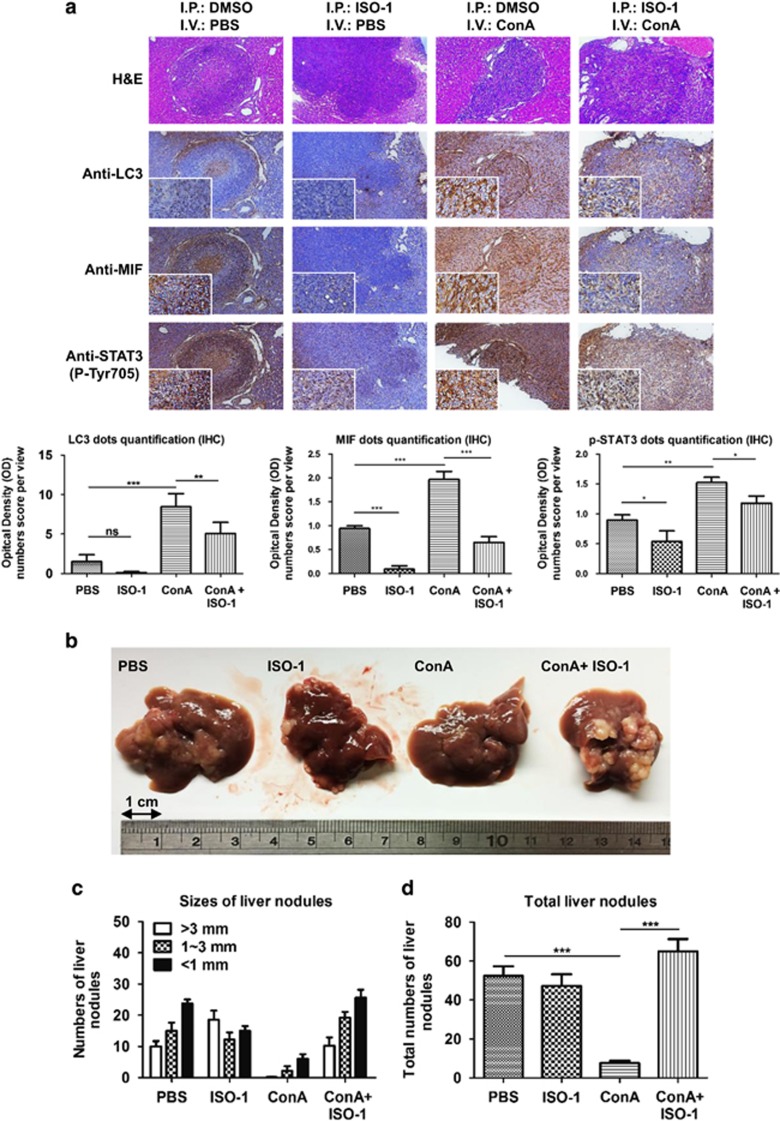
Suppression of MIF abrogates the therapeutic effect of ConA on hepatoma cells *in vivo*. First, 5 × 10^5^ ML-1_4a_ established tumor nodule formations were used for intrasplenic inoculation of BALB/c mice. After 7 days of tumor nodules formation, ConA (10 mg/kg) and PBS were given intravenously and ISO-1 (10 mg/kg) and 5% DMSO were given intraperitoneally twice at 3-day intervals as indicated. At day 30, mice were killed and their liver tissues were removed for analysis. (**a**) H&E staining of mice liver tissues reveals the regions with tumor nodules (blue) and the regions without non-tumors (red). IHC staining revealed LC3-, MIF- and STAT3 (Tyr705)-positive cells in tissues. All photos were taken under a microscope at × 100 magnification. Quantification of positive cells from at least three different same-size images of tissues were analyzed by using Image j software. (**b**, **c** and **d**) Liver tissues were removed to determine the sizes and number of tumor nodules (*N*=6). All data are presented as the means±S.D. from at least triplicate independent experiments; *P<0.05, **P<0.01, ***P<0.001, ns indicated no significance

To further investigate the roles of MIF in the anti-hepatoma activity of ConA *in vivo*, we used an *in situ* hepatoma model in BALB/c mice.^24^ Briefly, after 1 week of intrasplenic injection of the murine hepatoma cells ML-1, hepatoma cells will migrate to the liver and form tumor nodules. ConA was given intravenously with or without ISO-1 intraperitoneally. Immunohistochemical analysis showed that MIF expression was ~2-fold higher, LC3 expression increased significantly by ~4-fold and STAT3 phosphorylation was 1.5-fold higher in ConA-treated tumor lesions compared with the control groups (Figure 7). However, the inhibition of MIF by ISO-1 rescued ConA-induced MIF and LC3 expression but only slightly reduced STAT3 phosphorylation (Figure 7a and Supplementary Data). Last but not least, ConA treatment dramatically reduced both the number and size of liver tumor nodules compared with the control groups, and the therapeutic effect of ConA was abrogated in the presence of ISO-1 (Figures 7b–d). These results strengthened the finding that MIF indeed has an important role in ConA-induced autophagy and anti-tumor activities *in vivo*.

## Discussion

**Figure 8:**
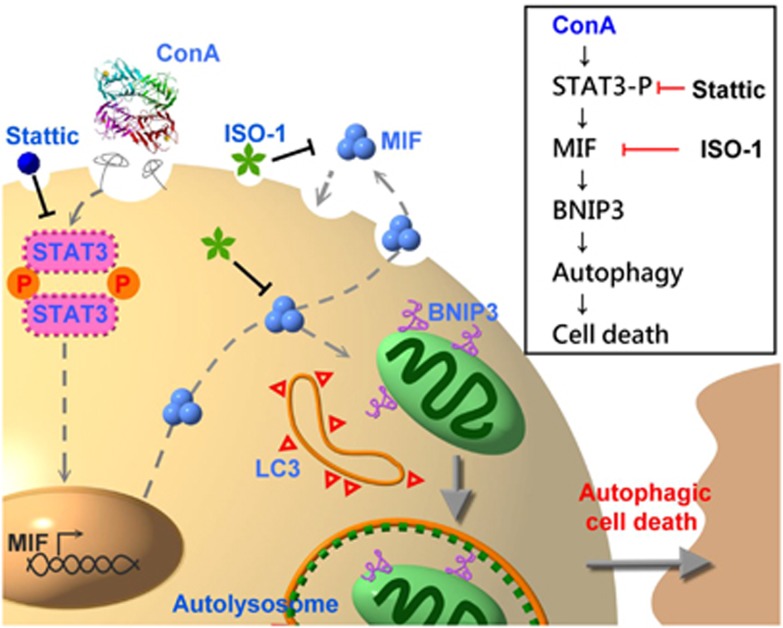
Hypothetical model of the signaling pathway through which MIF is involved in ConA-induced autophagic cell death of hepatoma cells. ConA internalization stimulates the phosphorylation of STAT3, which can increase MIF mRNA and protein expression/secretion. Binding of secreted MIF to hepatoma induces BNIP3 expression, which further induces LC3 accumulation to initiate autophagy and cause hepatoma cell death

In this study, we demonstrated that the ConA-induced autophagy of hepatoma cells was inhibited and cell death was rescued in the absence of MIF or the presence of MIF inhibitor. Therefore, MIF is required for the ConA-induced autophagic cell death of human hepatoma cells. A hypothetical model of the signaling pathway through which MIF is involved in ConA-induced autophagic cell death of hepatoma cells is shown in Figure 8. ConA internalization induces STAT3 activation, which in turn induces MIF expression and secretion. The increased MIF expression and secretion lead to autophagy through BNIP3 expression and LC3 conversion, which eventually causes hepatoma cell death. Furthermore, an *in situ* hepatoma murine model strengthened our hypothesis that MIF has an important role in ConA treatment by regulating autophagy.

Autophagic cell death, which is a non-apoptotic form of programmed cell death, has attracted considerable attention over the last few years due to its potential to kill tumor cells that are resistant to apoptosis.^46, 47^ Unlike apoptosis, autophagic cell death is a caspase-independent path that is associated with the appearance of double-membrane autophagosomes and dependent on autophagy proteins.^48, 49^ Although ConA-induced caspase-dependent apoptotic cell death has been reported in human melanoma A375 cells and reactivated astrocytes,^50, 51^ in this study we demonstrated that ConA induced a caspase-independent cell death in human hepatoma cells. In addition, the inhibition of autolysosome fusion by Baf A1 but not the caspase inhibitor Z-VAD significantly rescued ConA-induced cytotoxicity. Therefore, our results suggest that ConA preferentially induces autophagic but not apoptotic cell death of human hepatoma cells.

Previous studies have shown that ConA can induce the autophagic cell death of hepatoma cells in a BNIP-3-mediated manner. As ConA-immobilized beads have no effect on cell death, this process requires the internalization of ConA. After binding with a mannose moiety that resides on hepatoma cell membrane glycoproteins, ConA is internalized to the mitochondria via clathrin-mediated endocytosis to initiate autophagic cell death.^24, 52^ In this study, we further demonstrated that ConA internalization triggered STAT3 phosphorylation and the release of MIF, which is required for ConA to induce the autophagy and cell death of human hepatoma cells. It is known that on stimulation of receptor on cell surface, the phosphorylation of STAT occurs via the receptor kinase itself or indirectly by intermediary kinases such as JAK family. ConA-triggered STAT3 phosphorylation and BNIP3 induction requires a JAK/STAT signaling has been reported in mesenchymal stromal cells and glioblastoma cells.^44, 45^ However, further studies are required to understand whether or which JAKs are involved in ConA-mediated STAT3 phosphorylation of human hepatoma cells. On the other hand, evidence for the requirement of receptor endocytosis in STAT3 transcriptional activity has been documented. STAT3 phosphorylation alone is not sufficient for cytoplasmic STAT3 translocation to the nucleus, which requires receptor-mediated endocytosis.^53^ These may explain why ConA internalization is pre-requisite to trigger STAT3-mediated MIF transcription.

The precise mechanism of how MIF induces BNIP3 expression remains unclear. BNIP3, a member of the BNIP family, is an apoptotic protector that is responsible for the protection of virus-induced cell death. Similarly, MIF has been shown to upregulate Bcl-2 expression^54^ and has anti-apoptotic ability.^55, 56^ In addition, it has been reported that MIF can interact with BNIP2-like protein, which may be involved in the regulation of cell proliferation.^57^ The interaction of MIF and BNIP2-like protein may occur following uptake of MIF into target cells by non-receptor-mediated manner, which is one of the three hypothetic independent signal pathways for MIF.^58^ Therefore, we used rMIF to mimic MIF secretion induced by ConA-stimulated hepatoma cells, because MIF can bind to cells in a paracrine or autocrine manner. Interestingly, we found that rMIF induced BNIP3 expression and autophagy (Supplementary Data) but not STAT3 phosphorylation and cell death (data not shown). Therefore, other signals triggered by ConA in hepatoma cells are required to induce STAT3 phosphorylation and cell death, which remain to be investigated.

In our *in situ* hepatoma murine model, we demonstrated that the inhibition of MIF by ISO-1 abrogated the therapeutic effect of ConA in BALB/c mice. Thus, MIF has a crucial role in mediating ConA-triggered autophagic cell death both *in vitro* and *in vivo.* However, there are still some other factors in this study that need to be considered. First, as MIF is a multifunctional immune mediator that may also regulate other immune cells, we cannot rule out the possible involvement of other indirect immune responses in this immunocompetent mice model. Second, unlike what we found *in vitro*, we noticed that the phosphorylation of STAT3 in liver tumor nodules was slightly decreased in the presence of ISO-1 in an *in situ* murine model. A possible explanation for this discrepancy is that MIF may induce the production of other cytokines *in vivo*, which may also activate STAT3 phosphorylation. Using immunodeficient mice in a future study may further clarify the role of MIF in ConA-induced autophagic cell death *in vivo*. Nevertheless, MIF indeed is required for the ConA-triggered autophagic cell death of hepatoma cells *in vivo*.

In the past, MIF was commonly considered a crucial mediator of inflammation and auto-inflammatory diseases such as rheumatoid arthritis, sepsis and infection.^59, 60^ Over the years, MIF has been found to be highly expressed in many cancer cells and is correlated with tumor cell growth and even invasion and metastasis.^61, 62^ In the pathogenesis of the liver, MIF also contributes to HCC and liver cirrhosis.^63, 64^ Contrary to the pathogenic roles of MIF in previous studies, we found that MIF has beneficial and necessary roles in the ConA-induced autophagic cell death of hepatoma cells. Therefore, this study provides new insights into the biological function of MIF as an autophagy modulator in cancer therapy.

## Materials and Methods

### Cell lines

Human hepatoma cell line HuH-7 and Hep G2 cells were purchased from the Japanese Collection of Research Bioresources (Osaka, Japan) and American Type Culture Collection (ATCC, Manassas, VA, USA). The BALB/c hepatoma cell line ML-1 and HuH-7 ATG9 knockout cell line were generous gifts from Dr CP Hu (Veterans General Hospital, Taipei, Taiwan) and Dr Tamotsu Yoshimori (Department of Cellular Regulation, Research Institute for Microbial Diseases, Osaka, Japan), respectively. Cells were maintained in Dulbecco's modified Eagle's medium (DMEM) supplemented with 10% heat-inactivated fetal bovine serum (FBS, Hyclone, Logan, UT, USA). All cell cultures were maintained at 37 °C in 5% CO_2_ humidified incubator. Puro-resistant populations of stable clones, MIF, luciferase (Luc) and LC3 knockdown cells were performed using shRNA as previously described.^65^ In brief, lentiviruses were packaged and generated from shRNA plasmid (MIF: TRCN0000056818, Luc: TRCN0000072243 and LC3: TRCN0000154060; National RNAi Core Facility, Academia Sinica, Taipei, Taiwan), pMD.G and pCMVDR8.91 co-transfected HEK 293 T cells, which were transfected using HyFect (Leadgene Biomedical, Tainan, Taiwan) according to the manufacturer's instructions. Stable clones were infected with lentivirus and selected in culture medium containing Puro (2 *μ*g/ml).

### LDH release assay

A CytoTox 96 Non-Radioactive Cytotoxicity Assay Kit (Promega, Madison, WI, USA) was used to analyze LDH release and cytotoxicity according to the manufacturer's instructions. Briefly, HuH-7 and Hep G2 cells (8 × 10^3^ cells) were seeded on 96-well plates. Next, 2% FBS Phenol Red-free DMEM was refilled with treatment for distinct dose and time periods as indicated. Then, 50 *μ*l of supernatant was collected and mixed with 50 *μ*l of CytoTox 96 Substrate Reagent (CytoTox 96 Non-Radioactive Cytotoxicity Assay Kit) in each well. After 30 min incubation in the dark, 50 *μ*l of stop solution was added to each well. Finally, the absorbance was read at 490 nm using VersaMax microplate reader (Molecular Devices, Sunnyvale, CA, USA). LDH release percentage of control was calculated as [(OD_sample_−OD_control_)/OD_control_] × 100%.

### Sub-G1 phase analysis/fixed PI assay

To measure apoptosis, a fixed PI assay was performed. In brief, treated cells were resuspended and fixed with 70% ethanol at −20 °C overnight. Washed cells were incubated with DNA extraction buffer at 37 °C for 30 min and stained with PI/Triton X-100 buffer at room temperature for another 30 min in the dark. After the final wash, cells were analyzed using a FACScan flow cytometer (Becton, Dickinson and Company, Franklin Lakes, NJ, USA). The percentage of Sub-G1 phase was analyzed using the WinMDI 2.9 software (TSRI, La Jolla, CA, USA).

### pEGP-LC3, ptfLC3 punctae and immunofluorescent assay

For analysis of autophagy, HuH-7 cells were transfected with pEGP-LC3 or mRFP-GFP tandem fluorescence-tagged LC3 (ptfLC3) plasmid for 24 h. Transfected cells (4 × 10^4^ cells) were seeded onto 24-well plates. After incubation with treatment in culture medium for a specific time, the cells were washed with PBS, immobilized with 4% paraformaldehyde for 30 min and stained with Hoechst 33342 (2 *μ*g/ml) for another 10 min. The images were taken from a fluorescence microscope (Leica Geosystems AG, St Gallen, Switzerland) at a magnification of × 400 or an Olympus FV-1000 MPE confocal microscope (Olympus, Tokyo, Japan).

### Reverse transcription PCR

To analyze the mRNA levels, RT-PCR analysis was performed. Cells and tissues were homogenized and extracted by Tripure isolation reagent (Roche Molecular Biochemicals, Rotkreuz, Switzerland). Reverse transcription of total RNA was performed by using PyroRTase (Leadgene Biomedical). The specific primers used for RT-PCR were as follows: for human GADPH, 5′-AAGGTGAAGGTCGGAGTCAAC-3′ and 5′-GGGGTCATTGATGGCAACAATA-3′ for human MIF, 5′-CTCGAGCTGCAGGAAC CAATACCCAT-3′ and 5′-AAGCTTGGCATGATGGCAGAAGGACC-3′ for murine MIF, 5′-GAGGGGTTTCTGTCGGAGC-3′ and 5′-GTTCGTGCCGCTAAAAGTCA-3′ and for murine *β*-actin, 5′-CGGTTCCGATGC CCTGAGGCTCTT-3′ and 5′- CGTCACACTTCATGATGGAATTGA-3′. PCR was performed by using 2 × Taq PCR master mix (Leadgene Biomedical) and the samples were analyzed on a 1.5% agarose gel.

### MIF sandwich enzyme-linked immunosorbent assay

HuH-7 and Hep G2 cells (3 × 10^5^ cells) were seeded on six-well plates. The cells were incubated with treatment for distinct doses and time periods, as indicated in Figures 3b and c. For immobilized ConA treatment, beaded-agarose ConA (G-Biosciences, St Louis, MO, USA) were treated as indicated in Figures 6b and c. The supernatants were collected and the levels of secreted MIF were detected by human MIF sandwich enzyme-linked immunosorbent assay (MIF-ELISA) according to the manufacturer's instructions (R&D Systems, Minneapolis, MN, USA). The absorbance value at 450 nm was read by a VersaMax microplate reader.

### Western blotting

Whole-cell lysates were collected using RIPA buffer III (Bio Basic Inc., Markham, Ontario, Canada) and subjected to 12% SDS-PAGE for separation. Next, the gel was transferred onto a PVDF membrane (Pall, Ann Arbor, MI, USA) and blocked with 5% skim milk in TBST (0.05% Tween 20 in TBS). Primary antibodies against LC3 (MBL International, Woburn, MA, USA), phosphor Tyr 705-STAT3 (Cell Signaling Technology, Beverly, MA, USA), total STAT3 (Cell Signaling Technology), BNIP3 (Abcam, Cambridge, MA, USA), caspase 3 (Imgenex, San Diego, CA, USA), *β*-actin (Sigma-Aldrich, St Louis, MO, USA) or MIF rabbit polyclonal antibody purified from recombinant human MIF (rMIF)-immunized rabbit sera were incubated with membrane at 4 °C overnight and washed with TBST. HRP-conjugated goat anti-rabbit or rabbit anti-mouse IgG secondary antibodies (1 : 10 000 dilution; Leadgene Biomedical) were incubated for another 1 h and detected using an Enhanced Chemiluminescence Western Blotting Kit (Advansta, Menlo Park, CA, USA). The results of the western blotting were quantified using the Image J software (National Institutes of Health, New York, NY, USA).

### *In situ* hepatoma experiment of murine model

BALB/c mice (male, 8–10 weeks old) were purchased from National Laboratory Animal Center (Taipei, Taiwan). The murine *in situ* hepatoma model was generated as previously described.^24^ In brief, 5 × 10^5^ ML-1 cells were suspended in 100 *μ*l DMEM base and were intrasplenically injected into anesthetized mice (Pentobarbital, 50 mg/kg intraperitoneally). After 1 week, ML-1 migrated into the liver from the spleen and formed liver tumor nodules. Once liver tumor nodules formed 1 week after injection, ConA (10 mg/kg) and ISO-1 (10 mg/kg) were given intraperitoneally and intravenously, respectively. At the 30th day after intrasplenic injection, mice were killed and their livers were removed to perform immunohistochemistry (IHC) and determine the numbers and sizes of the tumor nodules.

### Hematoxylin and eosin stain and IHC

Fresh tissues were fixed with 4% formalin and embedded with paraffin. For hematoxylin and eosin (H&E) staining, biopsied sections (4 *μ*m) were stained with H&E as follows: Harris hematoxylin for 6 min, running tap water for 1 min, eosin Y for 10 min, 70% ethanol for 1 min, 95% ethanol for 1 min, 100% ethanol for 1 min and two rinses in 100% xylene for 1 min each. For IHC staining, the assay protocol followed the manufacturer's instructions (BIOTnA Biotech, Jongli City, Taiwan). In brief, after blocking with 0.3% hydrogen peroxide and 0.5% BSA, slides were incubated with primary antibody at 4 °C overnight. The primary antibody against LC3, phosphor Tyr 705-STAT3 or MIF rabbit polyclonal antibody from purified rMIF-immunized sera was incubated according to the manufacturer's protocol. After washing with PBS, the slides were incubated with secondary antibody (mouse/rabbit probe HRP labeling) for 30 min at room temperature and then loaded onto DAB mixed reagent for 5 min. Counterstain of hematoxylin was applied for 5 min.

### Statistical analysis

All statistical analyses were performed using the Prism software (GraphPad Software Inc., San Diego, CA, USA). The results were analyzed using unpaired Student's *t*-test, to compare the statistical data between two independent groups. For each result, all data are presented as the means±S.D. from triplicate independent experiments; *P<0.05, **P<0.01, ***P<0.001 and ns indicates no significance.

## Abbreviations

BNIP3: Bcl-2/adenovirus E1B 19 kDa-interacting protein 3
ConA: concanavalin A
ISO-1: (S,R)-3-(4-hydroxyphenyl)-4,5-dihydro-5-isoxazole acetic acid
MIF: macrophage migration inhibitory factor
STAT3: signal transducer and activator of transcription 3
