# Supplementary material for: Macrophage migration inhibitory factor has a permissive role in concanavalin A-induced cell death of human hepatoma cells through autophagy
Source: Cell Death Dis. 2015 Dec 3;6(12):e2008–. doi: 10.1038/cddis.2015.349 (PMC4720884; doi:10.1038/cddis.2015.349)
Supplement: Supplementary Information [file cddis2015349x1.docx]

**Supplementary data**

**
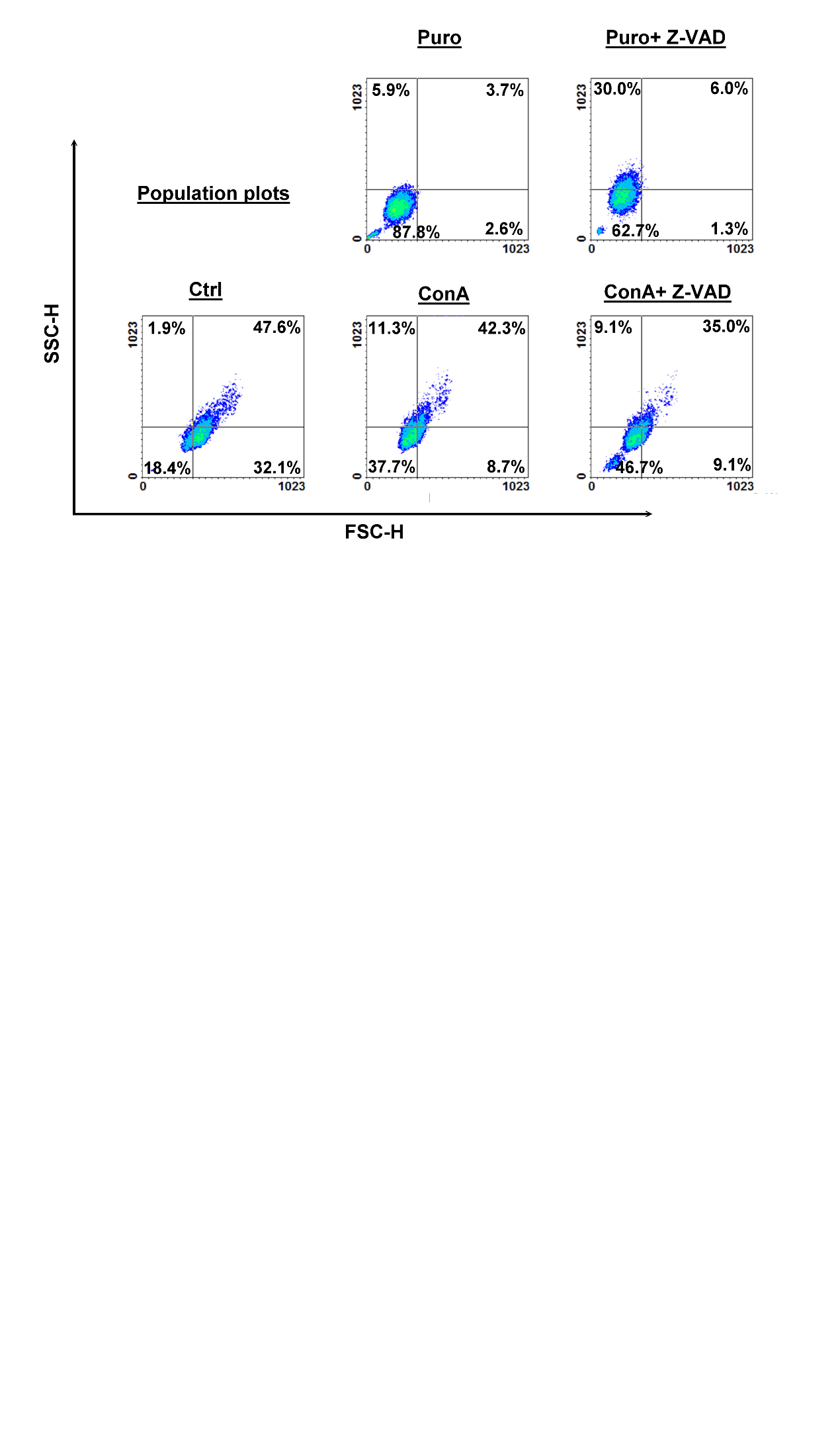
**

**S1.** **DNA fragment analysis of HuH-7 cells using flow cytometry.**

The experiment was performed as same as figure 2d. Total of 10,000 cells were collected and analyzed. A representative FSC/SSC plot of each condition was shown.

**
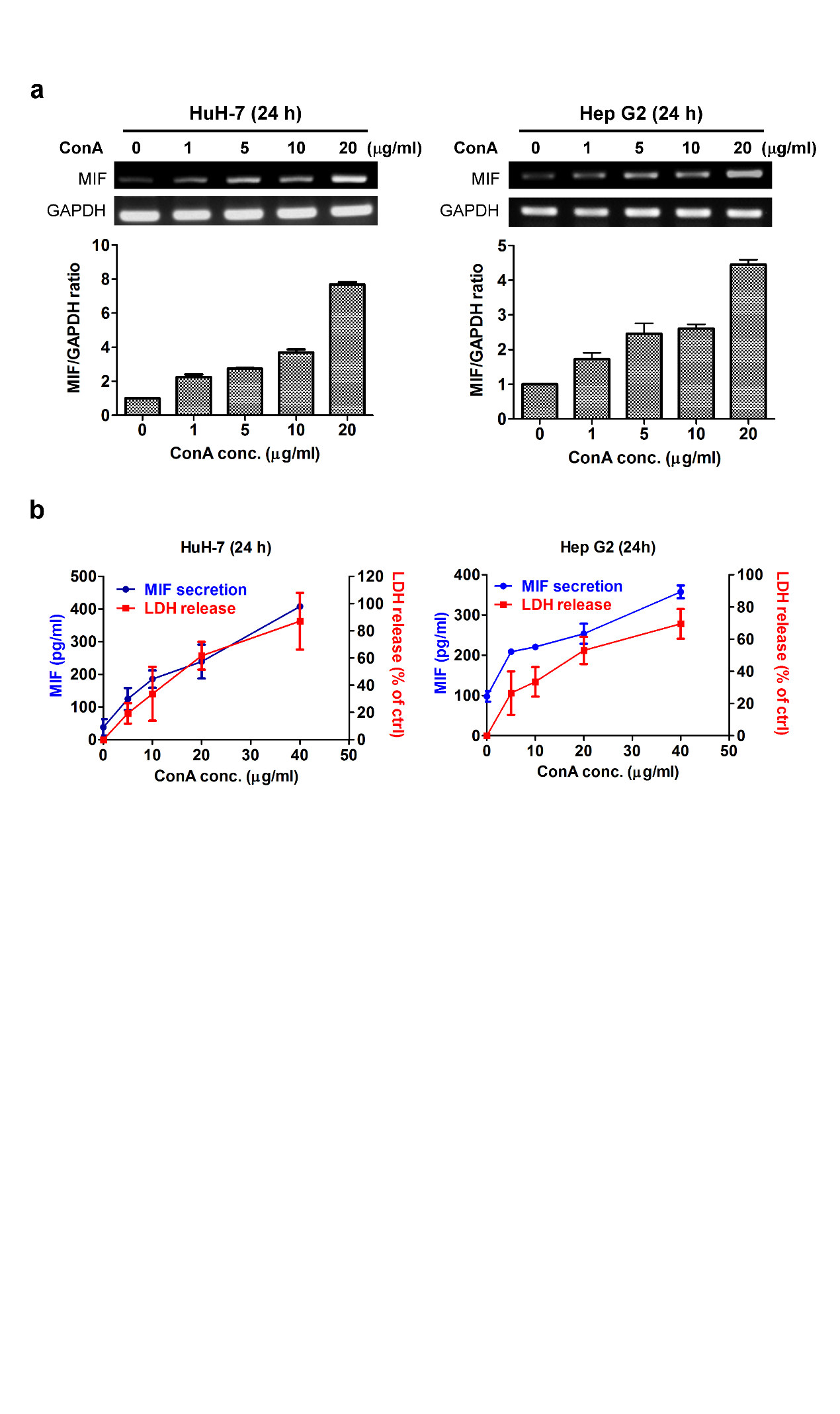
**

**S2. ConA upregulates MIF mRNA, protein, and secretion of human hepatoma cells.**

HuH-7 and Hep G2 cells were treated with the indicated doses of ConA for 24 h. (a) The MIF mRNA level was determined by RT-PCR. (b) The level of MIF secretion from culture supernatant was detected by MIF-ELISA (left y-axis). The cytotoxicity was analyzed by LDH release activity from culture supernatant (right y-axis). LDH release (% of ctrl) represents the percentage of LDH activity in media compared with control group. All data are presented as the mean±S.D. from at least triplicate independent experiments; *P<0.05, **P<0.01, ***P<0.001, ns indicates no significance.


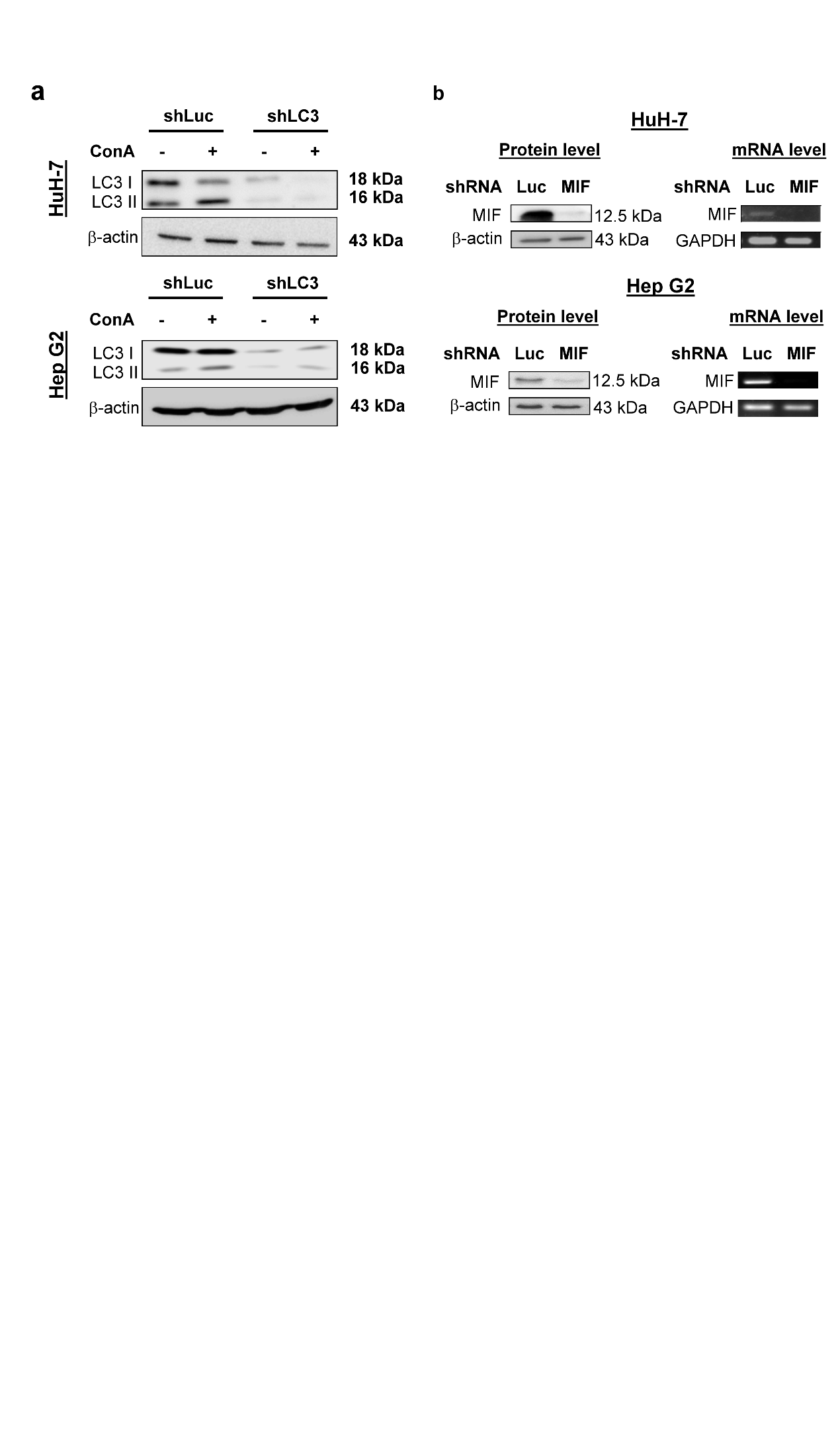


**S3. Knockdown of endogenous genes in human hepatoma cells.**

(a) HuH-7-shLuc/shLC3 and Hep G2-shLuc/shLC3 cells were treated with or without ConA (20 μg/ml) for 24 h. LC3 conversion was determined by western blotting. (b) HuH-7 and Hep G2 cells were used to perform MIF or Luc gene knockdown by shRNA. Protein and mRNA level of MIF were evaluated by western blotting and RT-PCR, respectively. All data are presented as the mean ±S.D. from at least triplicate independent experiments.


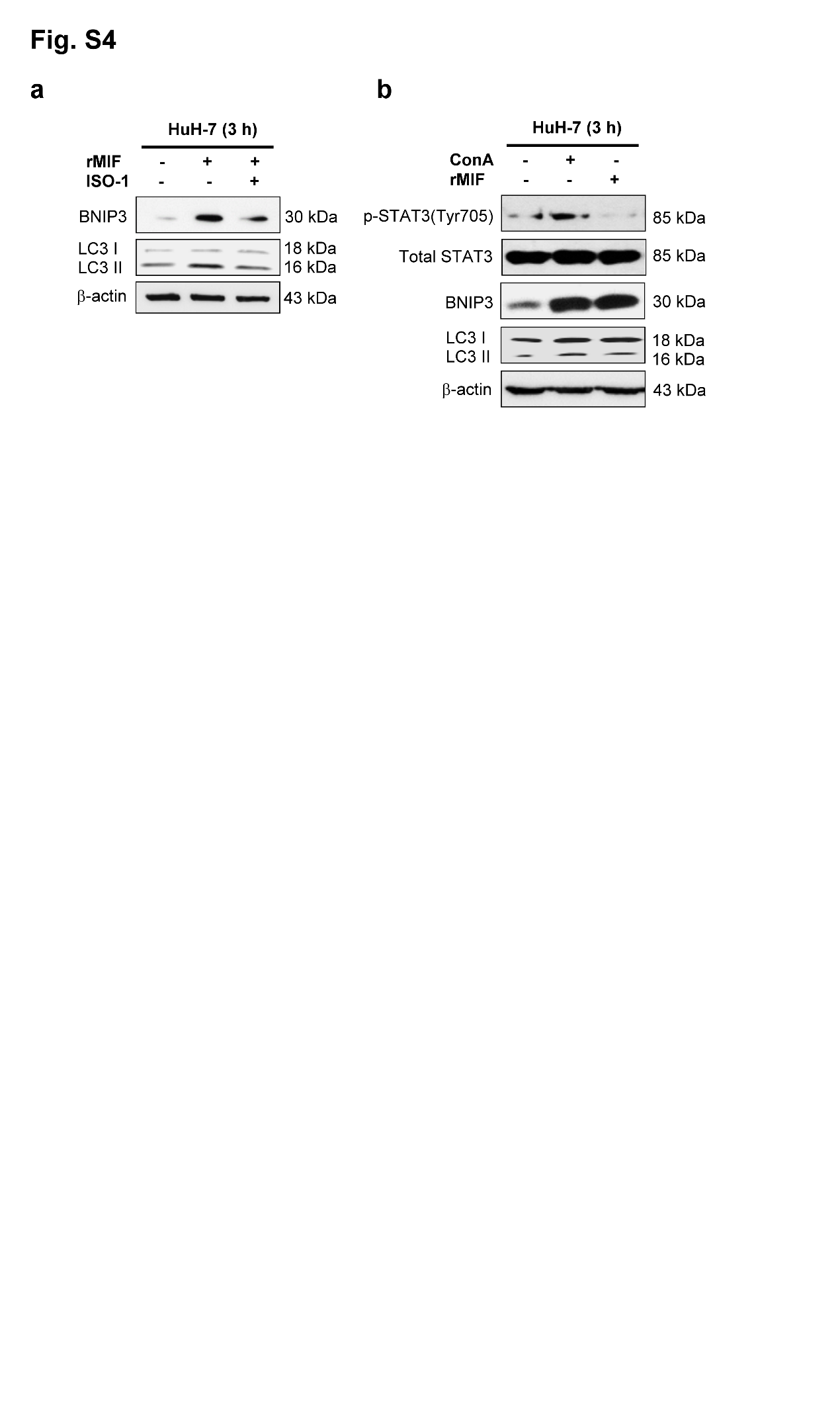


**S4. ConA and rMIF induce BNIP3 expression but only ConA triggers STAT3 phosphorylation.**

(a) HuH-7 cells were treated with rMIF (10 ng/ml) in the presence or absence of ISO-1 (50 μM) for 3 h. The lysates were subjected to detect BNIP3 expression and LC3 conversion by western blotting. (b) HuH-7 cells were treated with ConA (20 μg/ml) or rMIF (10 ng/ml) for 3 h. The lysates were subjected to detect STAT3 phosphorylation of Tyr705, total STAT3 and BNIP3 expression, and LC3 conversion by western blotting. All data are presented as the mean ±S.D. from at least triplicate independent experiments.


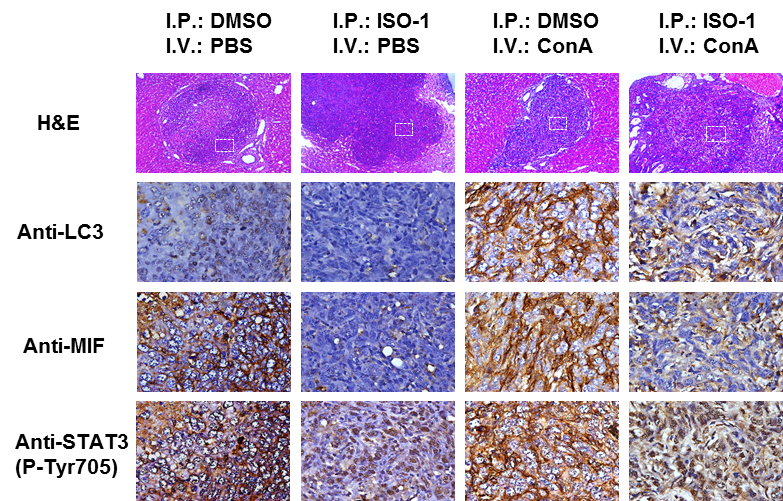


**S5. Suppression of MIF abrogates the therapeutic effect of ConA on hepatoma cells *in vivo*.**

Representative and enlarged images of H&E staining (× 100) and IHC analysis (× 400) of LC3, MIF, p-STAT3 in tumor nodules as same as figure 7.
